# Assessing Colorectal Care Capacity at an Urban Tertiary Hospital in Ghana

**DOI:** 10.5334/aogh.4663

**Published:** 2025-09-12

**Authors:** Kate V. Panzer, Antoinette A.A. Bediako-Bowan, Philemon Kumassah, Andrea Orji, Nathan R. Brand, Jonathan Dakubo, Pius T. Agbenorku, Samuel A. Debrah, Lyen Huang, Jonathan Laryea, Ann C. Lowry, Gifty Kwakye

**Affiliations:** 1 University of Michigan Medical School, Ann Arbor, Michigan, USA; 2 Department of Surgery, Korle Bu Teaching Hospital, Accra, Ghana; 3 Department of Surgery, University of Ghana Medical School, Accra, Ghana; 4 Department of Surgery, University of California, San Francisco, California, USA; 5 Department of Surgery, Kwame Nkrumah University of Science and Technology, Kumasi, Ghana; 6 Ghana College of Physicians and Surgeons, Accra, Ghana; 7 Department of Surgery, University of Cape Coast, Cape Coast, Ghana; 8 Department of Surgery, University of Utah, Salt Lake City, Utah, USA; 9 Department of Surgery, University of Arkansas for Medical Sciences, Little Rock, Arkansas, USA; 10 Department of Surgery, University of Minnesota, Minneapolis, Minnesota, USA; 11 Department of Surgery, University of Michigan, Ann Arbor, Michigan, USA

**Keywords:** global surgery, colorectal surgery, capacity assessment, global surgical education, ghana

## Abstract

*Background:* The burden of colorectal diseases continues to rise in Ghana. However, building a surgical workforce to address these diseases has been hampered by the lack of a colorectal specialty training pathway. To address this gap, the first colorectal surgery fellowship in Ghana was established in July 2023.

*Objective:* This study aims to identify strengths and gaps in colorectal care delivery prior to fellowship implementation by assessing relevant infrastructure, resources, and case volume at a Ghanaian teaching hospital.

*Methods:* Data on surgical infrastructure and human resources were collected at Korle Bu Teaching Hospital (KBTH) in Accra, Ghana. Retrospective, de-identified data were collected on all colorectal procedures performed at KBTH from January 1, 2022, to December 31, 2022. Cases were categorized by common anorectal, abdominal, and endoscopic procedures.

*Findings:* All surgical infrastructure and human resources were always available during the study period, except for immunohistochemistry services. 2,992 colorectal procedures were performed, including 173 anorectal procedures, 167 abdominal procedures, and 2,652 endoscopic procedures. The three most common colorectal surgeries performed were segmental colectomy (n = 76), excisional hemorrhoidectomy (n = 64), and stoma creation/management (n = 52). Some common colorectal services were not provided, including banding of internal hemorrhoids, seton placement for perianal fistulas, rectopexy for rectal prolapse, and pelvic floor evaluations.

*Conclusions:* There is a need for colorectal-specific surgical training and infrastructure in Ghana. KBTH is well-equipped with the resources to support growth of the newly established colorectal surgery fellowship, which will expand colorectal services available for Ghanaians.

## Introduction

The global burden of both benign and malignant colorectal diseases has risen in recent years, particularly in low- and middle-income countries (LMICs) [1–3]. Ghana is no exception to this trend, where colorectal cancer (CRC) has increased eight-fold since the 1960s [4, 5]. Despite the identification of CRC as a major priority by the Ghana Ministry of Health in 2011, the five-year survival rate following CRC diagnosis remains low at 16% [6, 7]. Surgery is an effective treatment for early-stage CRCs and many refractory benign colorectal diseases, but limited access to specialized surgical training is a barrier to care in LMICs [3, 8–10].

As a result, CRC in LMICs is often provided by general surgeons without colorectal-specific training [3]. Prior to 2023, no dedicated colorectal surgery training program existed throughout West Africa [11]. To address this significant gap, the first colorectal surgery fellowship was established in Ghana, with the inaugural fellow primarily stationed at Korle Bu Teaching Hospital (KBTH) [12]. KBTH, the largest tertiary care center in Ghana, is located in the capital city of Accra and serves as a referral center not only for Ghana’s population of 33 million but also for surrounding West African countries. This 2,000-bed hospital, which has grown from its original 192-bed capacity, now handles an average of 1,500 outpatient visits and 250 patient admissions daily, making it the largest hospital in West Africa and the third largest on the African continent. Given its central role in providing specialized care across the region, KBTH was chosen as the primary site for this fellowship. The fellowship curriculum was developed in collaboration with local and international partners, adapting requirements established by other colorectal surgery accrediting bodies to fit the LMIC context. For example, US-accredited colorectal surgery fellowships require minimum procedural volumes across major categories which provided a framework for identifying priority skills while adjusting expectations to the Ghanaian case mix and available resources [13]. However, the hospital’s capacity to support the growing demand for this specialized training has not yet been assessed.

Surgical capacity assessments are often used to identify healthcare gaps and ultimately improve surgical access and outcomes in resource-limited settings [14–16]. While surgical capacity in Ghana has been investigated for several specialties, literature on colorectal surgery in this setting is limited [17–19]. To determine what support is needed for the success of the colorectal surgery fellowship and to identify gaps in existing care delivery, this study aims to assess the surgical infrastructure, human resources, and colorectal operative volume at a tertiary care center in Ghana that existed prior to the establishment of the fellowship.

## Methods

Data on surgical infrastructure (e.g., hospital beds, operating room equipment, imaging modalities, laboratory capabilities) and human resources (e.g., surgeons, anesthesiologists, radiologists, pathologists) at KBTH were collected via the Surgical Oncology Assessment Tool (Table 1). This tool was adapted from the World Health Organization (WHO) Surgical Assessment Tool, and additional questions were added to include pathology [20]. These additions were developed via expert opinion from pathologists working in East Africa. Data were gathered by research investigators at KBTH via direct communication with department leadership. Approval was granted by the University of Michigan Institutional Review Board and the KBTH Institutional Review Board.

**Table 1:** Surgical oncology capacity assessment tool.

GENERAL SURGERY INFRASTRUCTURE		HUMAN RESOURCES	
General Infrastructure	Availability	Surgery	Quantity
Electricity/Power Generator Running Water Internet Oxygen # General Surgery Admissions per year # General Surgery Outpatients per year # Hospital Beds # General Surgery Beds # Post-Op Beds # Surgical ICU Beds # OR ventilators # ICU ventilators Pulse Oximetry General Anesthesia IV Sedation Anesthesia Spinal Anesthesia Regional Anesthesia	Always Always Always Always 2,915 9,926 1600 150 6 4 12 20 Always Always Always Always Always	Total # Surgical Providers # Surgeons # Medical Officers # Asst Medical Officers Ever >1 month without surgeon? Surgical provider availability # Surgical Nurses # Theatre Assistants	41 12 29 N/A No 24 hours/day 38 150
OR Equipment	Availability		
# Functional ORs^b^ # Anesthesia Machines Surgical Instruments Sterilizer Consumables	23 23 Always Always Always		
Pharmacy	Availability	Anesthesia	Quantity
Post-Op IV Narcotics Post-Op Oral Narcotics Antibiotics for Surgery IV Fluids for Surgery	Always Always Always Always	# Anesthesia Providers Ever >1 month without anesthesia provider? Anesthesia provider availability	16 No 24 hours/day
Radiology	Availability	Radiology	Quantity
Functional Ultrasound Functional X-ray machine Functional CT scanner Functional MRI scanner	Always Always Always Always	# Radiologists # Radiology Technicians Ever >1 month without radiologist?	12 17 No
Pathology and Laboratory Medicine	Availability	Pathology	Quantity
Laboratory in Facility Able to administer blood transfusions Biopsy services Biopsy approaches Staff who perform biopsies	Yes Always Yes CNB, EB, FNA Radiologists, General Surgeons	# Pathologists # Pathology Technicians Ever >1 month without a pathologist? # CNBs performed # EBs performed	3 10 No 275 84
Biopsy assessment location Turnaround time for biopsies Complete Blood Counts Chemistry Studies Coagulation Studies Histology Services Cytology Services Immunohistochemistry Services Barriers to the Above Services Annual frequency of equipment down Use of synoptic reporting templates	Onsite + private lab 1 week (private), 4 weeks (onsite) Always Always Always Always Always Sometimes Reagent stock outs None No (private lab uses synoptic reporting)		
Multimodal Care	Availability		
Administration of Chemotherapy Administration of Radiation Therapy	Always Always		

Key: Intensive Care Unit (ICU), Operating Room (OR), Intravenous (IV), Complete Blood Count (CBC), core needle biopsy (CNB), excisional biopsy (EB), fine needle aspiration (FNA).

^a^ ICU ventilators include 4 in surgery ICU, 6 in Medical ICU, and 10 in Pediatric ICU.

^b^ ORs include First-floor theatre (7: general surgery, urology, ENT, maxillofacial); pediatric surgery (2); obstetrics (2); gynecology (2); orthopedic (2); trauma (2); plastic surgery (2); cardiothoracic (2); neurosurgery (2).

^c^ Consumables include sterile gloves, hand wash, skin prep.

^d^ Theater assistants include surgical nurses, technicians, assistants.

To quantify colorectal service delivery, retrospective, de-identified data were collected on all colorectal surgery procedures performed at KBTH in Accra, Ghana, from January 1, 2022, to December 31, 2022. A one-year period was chosen to simulate the estimated case volume a fellow will be exposed to over the duration of their training. The date of surgery, postoperative diagnosis, and procedure performed were documented for each operative case. These data were consolidated to quantify the major types of colorectal procedures performed during the study period (Table 2). The identified surgical procedures were selected based on the minimum case requirements outlined by the Accreditation Council for Graduate Medical Education (ACGME) for US-accredited colorectal surgery fellowships (Table 3), along with expert opinion from colorectal surgeons in the USA and general surgeons in Ghana [13, 21]. These ACGME benchmarks provided a framework for identifying priority operative skills, while allowing adaptation to Ghana’s case mix, disease burden, and available resources. The availability of resources and presence of barriers were reported by general surgery faculty and resident investigators at KBTH.

**Table 2:** Colorectal service delivery assessment.

ANORECTAL PROCEDURES				
CONDITION	PROCEDURE	TOTAL	AVAILABILITY	BARRIERS
Anal Dysplasia	Fulguration of condylomas	4	Adequate	
	High-resolution anoscopy for anal dysplasia	0	Unavailable	Absent, Training
Anorectal Mass	Transanal excision (polyp, mass)	2	Adequate	
Fecal Incontinence	Overlapping sphincteroplasty	1	Inadequate	Personnel
Fistula	Seton placement for perianal fistula	0	Unavailable	Infrastructure
	Fistulotomy for perianal fistula	37	Adequate	
	Transsphincteric fistula repair (w/ plug, glues, or ligation of the intersphincteric fistula tract [LIFT], etc.)	13	Adequate	
	Endorectal advancement flaps	5	Adequate	
	Rectovaginal fistula repair with transposition flaps	4	Inadequate	Training
Fissure	Botox injections	0	Unavailable	Absent, Infrastructure
	Lateral internal sphincterotomy	7	Adequate	
Hemorrhoid	Excisional hemorrhoidectomy	64	Adequate	
	Banding of internal hemorrhoids	0	Unavailable	Other^a^
Rectal Prolapse	Perineal rectosigmoidectomy (Altemeier)	6	Adequate	
	Mucosal sleeve resection (Delorme)	0	Unavailable	Absent
**ABDOMINAL PROCEDURES**				
**CONDITION**	**PROCEDURE**	**TOTAL**	**AVAILABILITY**	**BARRIERS**
Bowel resection (for cancer/ masses, IBD, diverticulitis, infections, etc.)	Abdominoperineal resection	6	Adequate	
	Intersphincteric resection of the rectum	16	Adequate	
	Lower anterior resection	10	Adequate	
	Segmental colectomy	76	Adequate	
Crohn’s/Genetic disorder related surgeries	Ileal anal pouch procedures	2	Adequate	
	Strictureplasty	0	Unavailable	Other^b^
Rectal prolapse	Rectopexy	0	Unavailable	Training
Stoma	Stoma creation and management of complications	52	Adequate	
**ENDOSCOPIC PROCEDURES**				
	**PROCEDURE**	**TOTAL**	**AVAILABILITY**	**BARRIERS**
	Anoscopy	36	Adequate	
	Colonoscopy	336	Adequate	
	Rigid/flexible sigmoidoscopy	720	Adequate	
	Upper endoscopy	1560	Adequate	
**PELVIC FLOOR EVALUATION**				
	**PROCEDURE**	**TOTAL**	**AVAILABILITY**	**BARRIERS**
	Anorectal manometry	0	Unavailable	Infrastructure, absent
	Balloon expulsion	0	Unavailable	Infrastructure, absent
	Endorectal ultrasound	0	Unavailable	Infrastructure, absent
	Defecography	0	Unavailable	Infrastructure, absent
	Pudendal nerve terminal motor latencies (PNTML)	0	Unavailable	Infrastructure, absent
	Pelvic floor physical therapy	0	Unavailable	Training, Other^c^

^a^ Tool for banding of internal hemorrhoids was available but not used during the study period.

^b^ No referrals received from gastroenterology for a patient who required a stricturoplasty during the study period.

^c^ Pelvic floor physiotherapy is performed through the KBTH Department of Obstetrics and Gynecology.

**Table 3:** Minimum case numbers for US-accredited colorectal surgery fellowships *(adapted from Accreditation Council for Graduate Medical Education, 2017)* [13].

PROCEDURE CATEGORY	MINIMUM NUMBER	EXAMPLES
**Anorectal Procedures**	60	Hemorrhoidectomy (10 excisional), fistula surgery (30), internal sphincterotomy (2), transanal excision (10)
**Abdominal Procedures**	120	Segmental colectomy (50), laparoscopic resections (30), low anterior resection (20), abdominoperineal resection (5), ileal pouch procedures (5), prolapse repair (6 total: 3 abdominal, 3 perineal), stomas (20 total; 5 for complications)
**Endoscopy/Pelvic Floor**	185	Colonoscopy (140 total; 30 interventional), proctoscopy/anoscopy (30), pelvic floor evaluation (15)

## Results

This study found that all general infrastructure, operating room equipment, pharmacy, radiology, and pathology resources were always available during the study period except for immunohistochemistry services, which were only sometimes available due to reagent stockouts (Table 1). The hospital’s 23 operating rooms are organized into specialized complexes or pods: orthopedic (2), trauma (2), pediatric surgery (2), plastic surgery (2), obstetrics (2), gynecology (2), cardiothoracic surgery (2), neurosurgery (2), and a first-floor theater housing general surgery, ENT, maxillofacial, and urology (7). All theaters are equipped and remain functional year-round. Although daily utilization varies with case load and emergencies, each complex is consistently staffed, allowing for a steady flow of surgical cases.

The assessment of human resources at KBTH identified 41 surgical providers, including 12 attending general surgeons and 29 trainees rotating across surgical disciplines. Additional essential personnel included 38 perioperative surgical nurses, 150 theater assistants, 16 anesthesia providers, 12 radiologists, 17 radiology technicians, 3 pathologists, and 10 pathology technicians serving the entire hospital. Surgical and anesthesia providers were available around the clock. The 150 theater assistants—comprising general nurses, theater nurses, assistants, and orderlies—were distributed across the various theater complexes and organized into eight-hour shifts, ensuring continuous coverage.

From January 1, 2022, to December 31, 2022, general surgeons at KBTH performed a total 2,992 colorectal procedures. Of these, 340 were operative cases, comprising 173 anorectal and 167 abdominal procedures. Emergency presentations accounted for 62% (212/340), while 38% were elective. Residents served as primary surgeons in 45% of these cases, with the remainder led by attending surgeons. The additional 2,652 procedures were endoscopic. Specific procedure types were further categorized and reported (Table 2). The three most common colorectal surgeries performed were segmental colectomy (n = 76), excisional hemorrhoidectomy (n = 64), and stoma creation/management (n = 52).

Some specific colorectal services, as required by US-accredited colorectal surgery training programs, were not provided during the study period, highlighting significant gaps in care. These include seton placement for perianal fistulas, high-resolution anoscopy for anal dysplasia, botox injections for fissures, banding of internal hemorrhoids, mucosal sleeve resection, rectopexy for rectal prolapse, and strictureplasty for inflammatory bowel disease (IBD) and minimally invasive colorectal procedures. Other procedures were performed infrequently, including rectovaginal fistula repair with transposition flaps (n = 4) and overlapping sphincteroplasty for fecal incontinence (n = 1). Of note, no pelvic floor evaluations were performed by the general surgery team due to a lack of training. However, trained physiotherapists who perform pelvic floor physiotherapy for obstetric patients after delivery were available if needed for colorectal pathologies.

## Discussion

This study describes the availability of infrastructure and human resources specific to colorectal surgery at a tertiary hospital in Accra, Ghana, from January 1, 2022, to December 31, 2022. Nearly all surgical infrastructure (e.g., operating room equipment, medications, laboratory testing) and human resources (e.g., surgeons, anesthesiologists, radiologists) were always available at KBTH (Table 1), demonstrating reliable resources for the expansion of colorectal training and services at this facility. Additional data on colorectal operative volume were reported during the same period, identifying adequate provision of several procedures (e.g., segmental colectomy, excisional hemorrhoidectomy, stoma creation/management) while revealing the low volume or absence of other services (e.g., internal hemorrhoid banding, seton placement, rectopexy) (Table 2).

Despite KBTH’s size, there are only four surgical ICU beds. In Ghana, as in many sub-Saharan African countries, the critical care subspecialty is underdeveloped, and most hospitals do not have dedicated surgical critical care specialists. This limited critical care capacity may constrain postoperative management for complex colorectal patients and underscores the need to develop ancillary skills and training such as perioperative optimization and postoperative monitoring protocols that can support specialized surgical efforts even in the absence of robust ICU infrastructure.

Upper endoscopy (EGD) accounted for a significant portion of the endoscopic procedures performed, with 1,560 EGDs conducted, reflecting the high demand for these services. In contrast, only 336 colonoscopies were performed, which likely reflects the lack of widespread CRC screening protocols rather than actual population needs. While EGD is not traditionally classified as a colorectal procedure, its inclusion highlights the broader scope of practice required of surgeons in Ghana due to a severe shortage of gastroenterologists. This underscores the need to tailor graduate medical training programs in resource-limited settings to address local healthcare gaps, rather than adhering to predefined molds designed for resource-rich countries.

The limitation of surgical resources, infrastructure, and personnel is a commonly cited barrier to performing surgery in LMICs. One study on anesthesia care in 22 LMICs found that less than half of the surveyed hospitals had the capacity to perform general anesthesia [22]. Other surgical capacity assessments of hospitals in LMICs found similar limitations in anesthesia machines, pulse oximetry, and basic infrastructure needs (i.e., electricity, running water, oxygen) [14, 23–25]. Insufficient availability of trained personnel prohibits many hospitals from providing essential surgeries. For example, an analysis of surgical human resources in East Africa found a lack of surgeons and anesthesiologists among all eight surveyed sites [26]. Although a capacity assessment of 17 hospitals in Ghana reported consistent running water, electricity, and laboratory capabilities, only one-third of the surveyed hospitals had a fully trained surgeon [18]. Fortunately, KBTH was found to have consistent basic infrastructure and surgical resources in addition to several surgical providers, anesthesiologists, nurses, and theater assistants to provide a safe and reliable surgical environment for the expansion of colorectal services.

Historically, general surgeons exclusively provided colorectal care in Ghana. Limited data show early studies at KBTH reported 25 laparotomies annually between 1987 and 2007 [27, 28]. In 2014, KBTH surgeons began offering intersphincteric resection (ISR) of the rectum with colo-anal anastomosis for low-lying rectal cancers and performed 102 ISRs within the next 7 years [29]. Beyond CRC, investigation of fistula-in-ano at KBTH found that 124 fistula surgeries were performed in 2014–2021, with fistula tract ligation being the most common followed by fistulectomy and fistulotomy [30]. Although these studies demonstrate an increase in colorectal surgeries at KBTH, the case volumes likely still underestimate true demand. None of the general surgeons providing colorectal services had received formal training in the conduct of more specialized cases, with some relying on self-taught techniques from available online resources [12]. We anticipate that case numbers will rise as both referring physicians and the general population become more aware of the colorectal surgery expertise being established through the new fellowship program.

While many essential colorectal procedures were found to be adequately available at KBTH, the current study identified important gaps (Table 2). For instance, there are multiple surgical approaches for the treatment of rectal prolapse, including perineal rectosigmoidectomy (Altemeier), mucosal sleeve resection (Delorme), and rectopexy. Although six Altemeier procedures were performed during the study period, no Delorme or rectopexy procedures were performed due to a lack of training. Additionally, while the department was offered tools to perform banding of internal hemorrhoids, this procedure was not yet incorporated into practice. Lastly, pelvic floor evaluations were found to be absent within the colorectal department due to a lack of infrastructure and training. Prioritization of these procedures within the new colorectal surgery fellowship may help to expand the diagnosis and management of undertreated colorectal pathology in Ghana. This will require creative solutions such as short-term external rotations to international sites that offer hands-on training opportunities, once appropriate licensure has been obtained, and incorporation of virtual reality technology for remote operative coaching.

This study has several limitations. First, the findings may not be generalizable to other healthcare facilities in Ghana. KBTH is a well-resourced tertiary care center located in Accra, where infrastructure and human resources are more readily available compared to other parts of the country. These resources, coupled with a large patient population, make KBTH an ideal location for fellowship training. However, such facilities and resources may not be as accessible in rural or less-developed areas. Second, the study was limited by the exclusion of pediatric surgical cases, which may conceal the burden of unique colorectal pathologies and the corresponding need for management [31]. Additionally, the Surgical Oncology Assessment Tool used to assess resources quantifies the infrastructure needed for basic surgical care but fails to capture specialized equipment crucial for colorectal surgery. For instance, despite the presence of basic surgical instruments, KBTH lacks essential tools like pelvic retractors (e.g., St. Mark’s or Wylie retractors) and high-powered headlights, which are critical for pelvic and anorectal procedures.

Another limitation is the inability to fully capture unmet surgical needs. For example, although four rectovaginal fistula (RVF) repairs were performed during the study period, the data do not account for patients who may have presented with RVF but were either referred elsewhere or did not receive surgical intervention due to a lack of trained personnel. This gap in data makes it difficult to quantify the full demand for procedures that are underutilized or not performed. Future studies should aim to assess unmet needs by tracking referrals and documenting conditions that went untreated within the facility, offering a more comprehensive understanding of care gaps.

Building on these findings, information from this study is already guiding targeted curriculum elements for Ghana’s colorectal surgery fellowship. Actions implemented to date include skills workshops for procedural gaps (e.g., rectopexy, seton placement, pelvic floor evaluation), visiting faculty for complex case mentoring, and short-term observerships at high-volume international centers. The curriculum is further supplemented with carefully selected operative videos from freely available online resources, and fellows receive sponsored membership to ASCRS University, providing access to member-only educational content, webinars, and clinical practice guidelines.

Additional priorities identified through this process include both technology-driven and capacity-building efforts. One focus is leveraging augmented reality (AR) and virtual reality (VR) to support remote intra-operative coaching, expanding access to expert guidance across sites. Another is partnering with the Ghana College of Physicians and Surgeons to strengthen subspecialty training in hepatopancreatobiliary surgery, surgical oncology, and surgical critical care. In parallel, developing targeted supply and equipment lists for KBTH and other training hospitals will help ensure the essential tools are consistently available. These lists will also serve as a foundation for coordinated donation outreach, aligning contributions with the clinical and educational needs most critical to high-quality training and patient care. In conclusion, this needs-driven approach keeps fellowship development responsive to evolving service demands while strengthening the surgical ecosystem in Ghana and the wider West African region. Our study highlights the existing capacity and gaps in colorectal care at a tertiary hospital in Accra, providing a foundation to refine the fellowship curriculum. Ongoing evaluation will be essential to ensure graduates are prepared to meet patient needs and to guide broader implementation across Ghana and other LMICs.

## Data Availability

The data that support the findings of this study are derived from patient records at Korle Bu Teaching Hospital and contain sensitive information. De-identified, aggregate data may be available from the corresponding author on request and with permission from the Korle Bu Teaching Hospital Ethics Review Committee.
